# The GTPase κB-Ras is an essential subunit of the RalGAP tumor suppressor complex

**DOI:** 10.1016/j.jbc.2025.110460

**Published:** 2025-07-05

**Authors:** René Rasche, Lisa Helene Apken, Sonja Titze, Esther Michalke, Rohit Kumar Singh, Andrea Oeckinghaus, Daniel Kümmel

**Affiliations:** 1 Institute of Biochemistry, University of Münster, Münster, Germany; 2 Institute of Molecular Tumor Biology, University of Münster, Münster, Germany; 3 National Institute of Immunohaematology, ICMR, Mumbai, India; 4 Department Metabolism, Senescence and Autophagy, Research Center One Health Ruhr, University Alliance Ruhr & University Hospital Essen, University Duisburg–Essen, Essen, Germany

**Keywords:** GTPase, GTPase-activating protein, Ras protein, tumor suppressor gene, X-ray crystallography

## Abstract

κB-Ras1 and κB-Ras2 are small GTPases with noncanonical features that act as tumor suppressors downstream of Ras. *Via* interaction with the RalGAP (GTPase-activating protein) complex, they limit activity of Ral GTPases and restrict anchorage-independent proliferation. We here present the crystal structure of κB-Ras1 in complex with the N-terminal domain of RGα2. The structure suggests a mechanism of intrinsic GTP hydrolysis of κB-Ras1 that relies on a scaffolding function of the GTPase rather than on catalytic residues, which we confirm by mutational analysis. The interaction with RGα2 is nucleotide independent and does not involve κB-Ras1 switch regions, which establishes κB-Ras proteins as a constitutive third subunit of RalGAP complexes. Functional studies demonstrate that κB-Ras proteins are not required for RalGAP catalytic activity *in vitro* but for functionality *in vivo*. We propose that κB-Ras may thus act as a regulator of RalGAP localization and thereby control the Ras–Ral signaling pathway.

Small GTPases represent a superfamily of proteins that are involved in the regulation of a wide range of cellular processes, including signaling, intracellular transport, and organization of the cytoskeleton (1). They act as molecular switches by cycling between an inactive GDP-bound and an active GTP-bound state (2). Two loop regions, termed switch I and switch II, adopt distinct conformations depending on the bound nucleotide. Only the GTP-bound conformation enables interaction with effector proteins that mediate downstream functions. The transition from off to on states involves exchange of GDP with GTP, whereas inactivation is achieved by GTP hydrolysis to GDP. The intrinsic rates of nucleotide exchange and hydrolysis are generally slow, and regulatory proteins control the GTPase activation state (3). Guanine nucleotide exchange factors promote nucleotide exchange to the active GTP-bound form (4), whereas GTPase-activating proteins (GAPs) accelerate GTP hydrolysis to switch the GTPase off (5, 6, 7).

Many of the signaling GTPases of the Ras family are implicated in the development of cancer and have thus received much attention. The oncogenes H-, N-, and K-Ras were extensively studied and provide a blueprint for understanding GTPase function (8). The G-nucleotide is recognized by several G-motifs with highly conserved amino acids, including a “P-loop lysine” that interacts with phosphates, an asparagine that discriminates guanine from adenine nucleotides, and an aromatic residue in switch I that interacts with the guanine base by edge-to-face π–π stacking interactions (9). A catalytic glutamine residue in switch II promotes the intrinsic hydrolysis of GTP and participates in stimulated GTP hydrolysis by GAPs that employ an “arginine finger.” However, there are exceptions from this canonical mechanism. The Ras family GTPases Ral, Rap, and Rheb are inactivated by “asparagine thumb” GAPs, which does not require contribution of a glutamine from the GTPase (7, 10, 11, 12, 13). Furthermore, the catalytic glutamine is not conserved in Rap GTPases, yet they show intrinsic GTP hydrolysis.

Other noncanonical GTPases of the Ras family are κB-Ras1 and κB-Ras2, which were originally identified as regulators of NF-κB signaling (14, 15). In contrast to most GTPases, they lack a membrane targeting motif and a catalytic glutamine. κB-Ras GTPases not only show intrinsic GTP hydrolysis but also have fast nucleotide exchange rates, which shifts them into a constantly active form at physiological G-nucleotide concentrations (16, 17).

The κB-Ras isoforms were shown to have redundant functions in counteracting the Ras–Ral signaling axis *via* the RalGAP complexes (18, 19). κB-Ras double knockout resulted in increased anchorage-independent proliferation and enhanced xenograft growth (17). In a genetically engineered mouse model, loss of κB-Ras in the pancreas of mice impaired function of the RalGAP complexes and led to elevated Ral GTP levels, which promoted tumor progression in a Ras-driven model of pancreatic ductal adenocarcinoma (20). κB-Ras binds RalGAP in a nucleotide-independent fashion, but how this binding impacts RalGAP function remains unclear (16). How κB-Ras exerts tumor suppressor function on a mechanistic level remains a fundamental question.

A cryo-EM structure of RalGAP showed that the complex contains an obligatory heterodimer of one of two α subunits (RGα1 and RGα2) with a catalytic asparagine thumb GAP domain and a β subunit (RGβ) that is essential for stabilizing the complex (21). RGα and RGβ interact *via* their C-terminal domains and form a tetramer *via* a homodimerization motif in the RGβ N-terminal domain (Fig. 1*A*). The κB-Ras binding site was mapped to the N-terminal domain of RGα (18), which, surprisingly, is far away from any region with known relevance for the RalGAP mechanism and was not resolved in the EM density.

**Figure 1:**
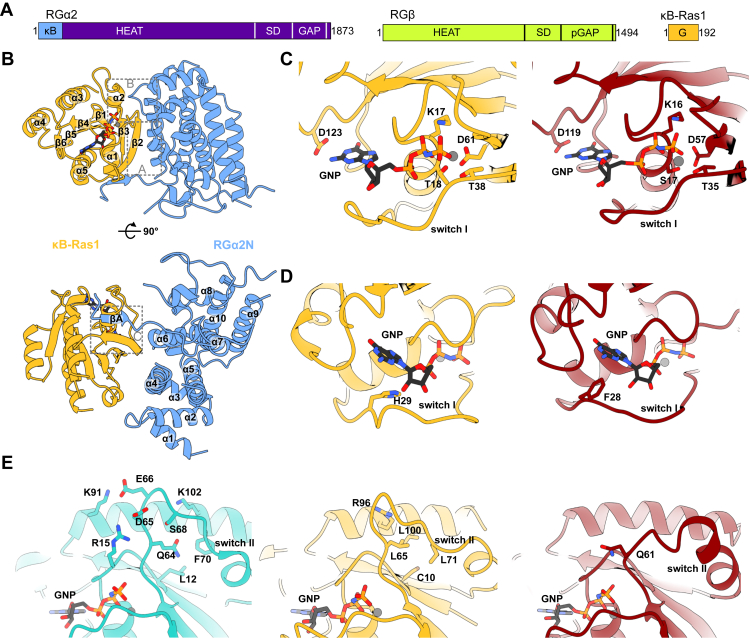
**Structural analysis of the κB-Ras1–RGα2N complex.***A*, domain organization of RalGAP subunits. G, G-protein domain; HEAT, HEAT repeat domain; κB, κB-Ras binding domain; (p)GAP, (pseudo-) GTPase-activating protein domain; SD, stabilization and dimerization domain. *B*, crystal structure of the κB-Ras1–RGα2N complex. κB-Ras1 (*yellow*) adopts a canonical G-domain fold, and RGα2N (*blue*) is composed of 10 α-helices that form a solenoid structure. Three main interaction sites are marked by *dashed boxes*. *C*, close-up of the nucleotide-binding pocket of κB-Ras1 (*yellow*) and H-Ras (*red*) with key residues shown in *stick representation*. *D*, close-up of the switch I region of κB-Ras1 (*yellow*) and H-Ras (*red*, Protein Data Bank ID: 5P21 (27)) with nucleotide-binding residues shown in *stick representation*. *E*, close-up of the switch II region of Rheb (*cyan*, Protein Data Bank ID: 1XTR (25)), κB-Ras1 (*yellow*), and H-Ras (*red*) with key residues shown in *stick representation*.

We therefore determined a crystal structure of κB-Ras in complex with the binding domain of RGα2 to obtain insight into the molecular function of κB-Ras. The structure of the subcomplex allows us to provide the first model of the intact RalGAP complex, and our functional analysis suggests a role of κB-Ras as a constitutive regulatory subunit of RalGAP. Elucidating the role of κB-Ras in RalGAP function sheds light on the critical Ral pathway in oncogenic Ras signaling.

## Results

### Crystal structure of κB-Ras bound to the RG**α**2 N-terminal domain

We sought to gain insight into the mechanism of κB-Ras protein function by structural analysis. Because κB-Ras was not resolved in the cryo-EM reconstruction of the RalGAP complex and did not crystallize when purified alone, we attempted structure determination of a κB-Ras1–RGα2 subcomplex. It was previously shown that κB-Ras GTPases interact with the N-terminal portion of the α-solenoid domain of the RGα subunits (18). We identified a fragment of RGα2 (RGα2N, residues 2–255) that forms a stable complex with κB-Ras1 (1–175) when coexpressed in bacterial cells (16). This RalGAP subcomplex was crystallizable, and we determined its structure at 2.72 Å resolution (Table S1, Figs. 1*B*, S1, *A* and *B*). κB-Ras1 adopts a canonical G domain fold comprised of a six-stranded central β-sheet and five α-helices (Fig. 1*B*). The N-terminal domain of RGα2 is composed of 10 helices forming tandem repeats that are twisted and have large loop insertions with short β-strand and α-helical elements. Two RGα2N molecules form a tail-to-tail dimer in the crystal unit cell (Fig. S1*C*). This is mediated by a hydrophobic interface, which is not functionally relevant as it is only exposed because the used RGα2^N^ construct was artificially truncated for bacterial expression.

### Canonical and noncanonical structural features of **κ**B-Ras

The nucleotide-binding pocket of κB-Ras1 is occupied by a magnesium ion and the nonhydrolyzable GTP analog 5′-guanylyl imidodiphosphate (GNP) (Figs. 1, *B* and *C* and S1*B*). The ligands are bound *via* conserved G-motif residues (Fig. S2), including Mg^2+^ coordination by Asp61 (Asp57 in Ras), phosphate interactions with the P-loop Lys17 (Lys16 in Ras), and hydrogen bonds of Asp123 with the guanine base (Asp119 in Ras). By interacting with the Mg^2+^ ion and Lys17, the β- and γ-phosphate are fixed in an eclipsed conformation (22). As observed for other Ras family proteins, the nucleotide γ-phosphate makes interactions with the backbone amides of the switch residues Thr38 in switch I (Thr35 in Ras) and Gly64 in switch II (Gly60 in Ras) (Fig. 1*C*).

However, the remainder of the switch regions in κB-Ras1 are strikingly different from those in other GTPases. First, switch I contains no aromatic residue capable of edge-to-face π–π stacking interactions with the guanine base (Phe28 in Ras). At the equivalent position, residue His29 of κB-Ras1 is oriented into the nucleotide-binding pocket and interacts with the GNP ribose 2′-hydroxy group. Consequently, switch I does not cover the nucleotide-binding pocket as closely in κB-Ras1 as in other GTPases (Fig. 1*D*). Furthermore, the two copies of κB-Ras1 in the asymmetric unit of the crystal show different conformations of switch I (Fig. S1*D*). Taken together, these observations indicate that the nucleotide-binding pocket of κB-Ras1 is destabilized, which provides a structural explanation for why the nucleotide affinity of κB-Ras1 is orders of magnitude lower than for most small GTPases (16).

Second, κB-Ras1 contains a leucine at position 65 instead of the catalytic glutamine 61, which is found at the equivalent position in Ras GTPases (Fig. 1*E*). Leu65 is tightly bound in a hydrophobic pocket formed by Cys10 of the β1-strand, Leu71 of switch II, and Leu100 of the α2-helix. Switch II is further stabilized by backbone interactions with Arg96 of helix α2, which collectively fixes switch II in a defined conformation. All positions that are relevant for nucleotide binding and switch II stabilization are conserved between κB-Ras1 and κB-Ras2 (Fig. S2).

### Intrinsic GTP hydrolysis by **κ**B-Ras

This fixed conformation of switch II in κB-Ras1 is reminiscent of the structure of the small GTPase Rheb (Fig. 1*E*). Although Rheb has the conserved Gln residue (Gln64) in switch II, this residue is not involved in intrinsic or GAP-stimulated GTP hydrolysis of Rheb (12, 23, 24). In the structure of GTP-Rheb (25), Gln64 occupies a similar position as Leu65 does in κB-Ras1. The conformation of Rheb switch II is primarily stabilized by polar interactions between switch II Asp65 and Asp66 with the P-loop Arg15 and Lys91 in helix α2, respectively, and backbone interactions of Lys102. Switch II conformation of Rheb is therefore largely independent of the bound nucleotide (25). Likely, switch II of κB-Ras1 will also adopt similar conformations in the GTP- and GDP-bound states.

**Figure 2:**
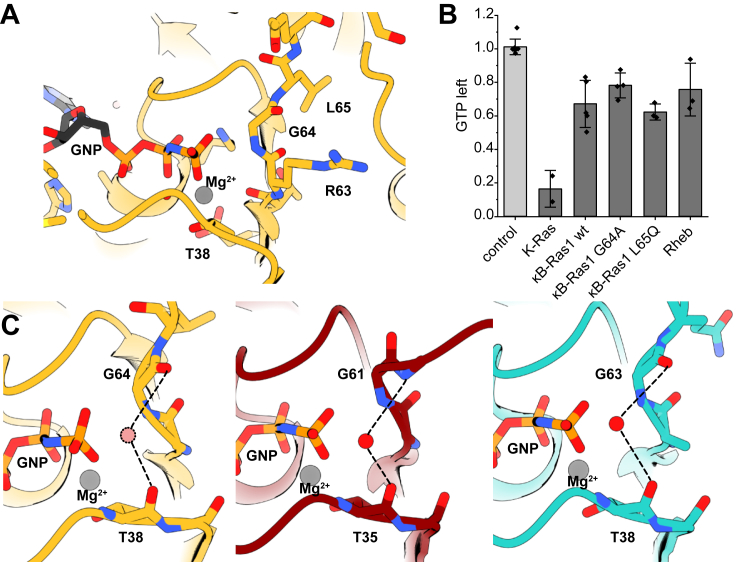
**GTP hydrolysis by κB-Ras1.***A*, interactions of κB-Ras1 switch I and switch II with the nucleotide γ-phosphate. *B*, intrinsic GTP hydrolysis by K-Ras, Rheb, κB-Ras1 WT, and variants measured *in vitro*. Data from independent repeats are shown as means ± SD. *C*, coordination of a hydrolytic water by switch I and switch II in κB-Ras1 (*yellow*) and H-Ras (*red*, (27)) and Rheb (*cyan*, (25)). For κB-Ras1, the water molecule (*dashed circle*) was placed in analogy to the water observed in the crystal structure of H-Ras (Protein Data Bank ID: 5P21 (27)).

Switch II residues of κB-Ras1 closest to the nucleotide γ-phosphate are Arg63, Gly64, and Leu65 (Fig. 2*A*). We previously showed that a κB-Ras2^R63A^ variant displayed a slightly reduced activity but was still capable of efficiently hydrolyzing GTP (16). To test if the switch II Gly64 is required for the intrinsic GTP hydrolysis activity of κB-Ras1, we generated the mutant κB-Ras1^G64A^. This substitution did not reduce intrinsic GTP hydrolysis activity of κB-Ras1 *in vitro* (Fig. 2*B*). We also generated a variant κB-Ras1^L65Q^, reintroducing the catalytic residue that was described for canonical Ras GTPases. However, GTP hydrolysis was also not altered by this mutation (Fig. 2*B*). Thus, the activity of κB-Ras proteins appears to be largely sequence independent, indicating that nucleotide binding and the protein fold may be sufficient to promote hydrolysis. This observation is consistent with previous work showing that, in principle, GTPase activity does not require a general base (26), and κB-Ras is thus an example for this mechanism.

We suggest that κB-Ras may exert a scaffolding function. As a result of κB-Ras1 L65 being pulled away from the nucleotide-binding pocket, the peptide bond to the preceding glycine 64 is flipped in comparison to H-Ras (Fig. 2*C*). This observation may provide a structural explanation for how κB-Ras proteins can hydrolyze GTP. The Gly64 carbonyl is oriented in a way that together with the backbone carbonyl of switch I Thr38, a water molecule could be coordinated in a position suitable for nucleophilic attack on the γ-phosphate of GTP. The limited resolution of the crystal structure does not allow us to confidently place water molecules in the experimental map, but the geometry and space in the active site would be consistent with water binding at the equivalent position as described for H-Ras (27) and also observed in Rheb (25). Notably, the catalytic water is coordinated by a backbone amide and a carbonyl in H-Ras, whereas coordination in Rheb and potentially κB-Ras1 involves two carbonyls (Fig. 2*C*). Thus, the nucleophilicity of a bound water in Rheb and, by analogy, κB-Ras1 would be higher, which could be a substitute for the requirement of a polarizing amino acid side chain at the active site. The similar conformation of switch II in κB-Ras1 and Rheb (Fig. 2*C*) may represent a homologous strategy for intrinsic GTP hydrolysis without catalytic glutamine. Of note, the efficiency of GTP hydrolysis by κB-Ras1 and Rheb is comparable (Fig. 2*B*).

### Interaction of **κ**B-Ras1 and RG**α**2

**Figure 3:**
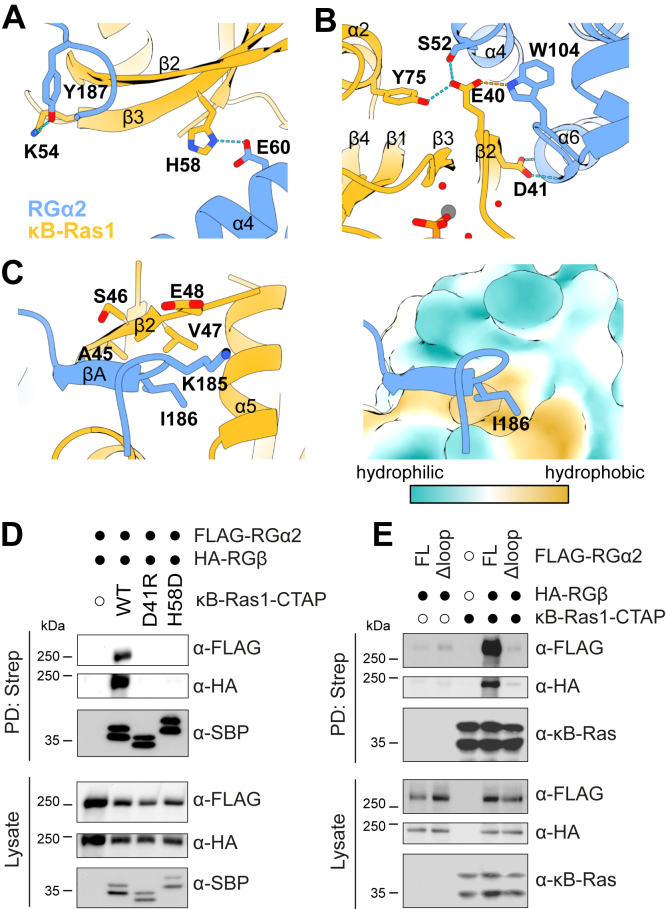
**Mutational analysis of the κB-Ras1–RGα2N interface.***A*, interaction of κB-Ras1 β3 with RGα2N helix α4 and the α8/α9 loop. *B*, interaction of κB-Ras1 β2 with RGα2N helix α6. *C*, binding of a short β-strand in the α8/α9 loop of RGα2N with a hydrophobic pocket on κB-Ras1. Close-ups correspond to the regions marked in Figure 1*B*. *D*, coimmunoprecipitation of RGα2/β by κB-Ras1 WT and variants. *E*, coimmunoprecipitation of RGα2 or RGα2Δloop with RGβ by κB-Ras1 WT and variants.

We observe no interactions of the κB-Ras1 switch region residues with RGα2N, consistent with previous findings that κB-Ras binding to RGα is nucleotide independent (16). The binding interface is constituted of helices α4 and α6 of RGα2N 10-helix bundle and the strands β1 and β2 of κB-Ras1 at the back side facing away from the nucleotide-binding pocket (Fig. 1*B*). Specific interactions are formed between RGα2N^E60^ and κB-Ras1^H58^ (Fig. 3*A*). In addition, κB-Ras1^D41^ interacts with the peptide backbone at the N-terminal end of RGα2 helix α6 (Fig. 3*B*). Finally, a large loop inserted between α8 and α9 of RGα2 (V157–F211) folds back into a binding groove between α1 and β2 of κB-Ras1 (Figure 1, Figure 3*B* and 3*C*). Here, the interaction occurs *via* β-sheet complementation and association of RGα2N^I186^ with a hydrophobic pocket on κB-Ras. The RGα2N-interacting residues are not conserved among Ras family GTPases (Fig. S2).

To validate the observed interactions and generate tools for functional studies, we designed mutations in κB-Ras that might disrupt RGα2 binding. The mutations κB-Ras1^H58D^ and κB-Ras1^D41A^, which map to the interface with α4 and α6 of RGα2 (Fig. 3, *A* and *B*), respectively, abolished κB-Ras binding to the RalGAP complex in coimmunoprecipitation (co-IP) studies (Fig. 3*D*). We also exchanged residues lining the hydrophobic pocket that accommodates Ile186 of RGα2 (Fig. 3*C*) to bulkier and more hydrophilic amino acids to block the pocket. Indeed, κB-Ras1^A45Q^ and κB-Ras1^V47D^ failed to bind RGα2/β in co-IPs, whereas κB-Ras1^S46Y^ had no effect (Fig. S3), possibly because it is located more at the periphery of the binding site. Expression levels of κB-Ras1^A45Q^ and κB-Ras1^V47D^ were reduced compared with WT, indicating that protein stability might be impaired. To exclude that the observed loss of interaction was a secondary effect caused by a structural defect in these variants, we also generated an RGα2 deletion construct RGα2Δloop, which lacks the relevant interacting portion of the α8/α9 loop (residues 178–189). In co-IPs, κB-Ras1 formed a complex with RGα2fl/RGβ, but not with RGα2Δloop/RGβ (Fig. 3*E*), demonstrating that this loop is required for recruitment of κB-Ras1 into RalGAP complexes. This mutational analysis confirms the interaction of RGα2 with an interface that does not include the switch regions of κB-Ras1 and is thus independent of the nucleotide loading status of the GTPase.

### Functional role of **κ**B-Ras

**Figure 4:**
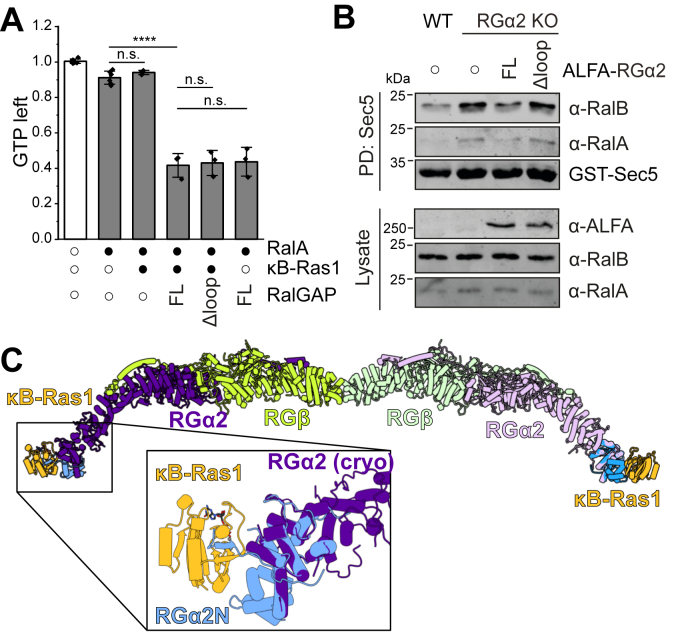
**Functional role of κB-Ras1 for RalGAP activity.***A*, *in vitro* Ral GAP assay with dimeric RGα2/β and trimeric RGα2/β/κB-Ras1 complexes. Data from independent repeats are shown as means ± SD, statistical analysis by one-way ANOVA. *B*, detection of cellular Ral activity levels by Sec5 pull-down in RGα knockout and reconstitutions with RGα2 or RGα2Δloop. *C*, composite model of the entire RGα2/β/κB-Ras1 from the superposition of the κB-Ras1/RGα2N crystal structure and the cryo-EM reconstruction of RGα2/β. GAP, GTPase-activating protein.

We next asked if binding of κB-Ras proteins to RGα influences GAP activity of the complex. Using an *in vitro* GAP assay, we show that the dimeric RGα2–RGβ complex stimulates the GTP hydrolysis by Ral to the same extent as the trimeric RGα2/RGβ/κB-Ras1 complex (Fig. 4*A*). Because the complexes were expressed in mammalian cells, endogenous κB-Ras may copurify with RGα2/RGβ. To exclude this possibility, we purified a κB-Ras binding deficient RGα2Δloop/RGβ complex. This also did not influence GAP activity (Fig. 4*A*), demonstrating that κB-Ras does not contribute to the catalytic mechanism of RalGAP.

However, elevated levels of active Ral have been reported in κB-Ras knockout cells (20). We thus tested the functional relevance of the κB-Ras1/RGα2 interaction in a cellular setting using RGα2 knockout cells. RalA and RalB are hyperactive in these cells, and Ral activity can be attenuated by reintroducing RGα2fl (Fig. 4*B*). In contrast, reconstitution with RGα2Δloop did not reduce levels of active Ral (Fig. 4*B*). Thus, the interaction of RGα2 with κB-Ras is required for RalGAP activity in cells, and the effect of κB-Ras on Ral signaling occurs directly and likely exclusively *via* RGα/β.

With the crystal structure of κB-Ras1/RGα2N described here and the previously published cryo-EM model of RGα2/RGβ, we are able to assemble a model of the entire RalGAP complex (Fig. 4*C*). The proteins form an extended hexameric assembly of ∼63 nm length. At the center, two RGβ subunits homodimerize with their N-terminal domains. RGβ and RGα2 interact *via* their C-terminal domains, positioning the N-terminal interaction site of RGα2 with κB-Ras1 at opposing ends of the complex. Thus, κB-Ras binds far away from the catalytic site on RGα2, consistent with the finding that κB-Ras does not contribute to the GAP mechanism of the complex.

## Discussion

The structural and biochemical data showed that κB-Ras is an integral part of the RalGAP complexes *via* a nucleotide-independent interface. κB-Ras does not contribute to the catalytic mechanism, but its stimulatory effect on RalGAP activity depends on cellular context. In the context of RalGAP architecture, κB-Ras is positioned at opposing ends like “feet” of an arch-shaped structure. We speculate that κB-Ras may play a role in recruiting RalGAP to the appropriate subcellular compartment(s), where the Ral GTPases need to be deactivated. Because different isoforms of RGα and κB-Ras are expressed, this opens up the possibility that different RalGAP complexes with specialized functions and distinct regulation exist in cells.

The interaction partner or receptor for RalGAP complexes on targeted compartments is currently elusive. κB-Ras proteins do not contain a lipidation motif, making direct membrane binding of RalGAP *via* kB-Ras unlikely. But because RGα is not an effector of κB-Ras, the switch regions of κB-Ras would be available to interact with a putative binding partner that mediates targeting of the complex. The search for an interactor that binds κB-Ras in an effector mode will be an important next step for understanding the regulation of the Ral–RalGAP signaling module.

The investigation of κB-Ras has also elucidated a new facet in the diversity of mechanisms of GTPase function. κB-Ras proteins are permanently in a GTP-bound (on) state, not because of their low intrinsic GTP hydrolysis rate or GAP insensitivity, but because they are fast-exchanging GTPases (16). Nucleotide affinity is reduced compared with canonical Ras GTPase but still below intracellular GTP concentration. A high intrinsic nucleotide exchange rate and low intrinsic GTP hydrolysis rate therefore render κB-Ras GTPases constantly active.

This may be the most effective way to achieve permanent GTP loading of a GTPase. We observe that despite the lack of a catalytic glutamine or other activating residues, κB-Ras hydrolyzes GTP (22). It was proposed that positioning of the catalytic water and occlusion from the solvent was sufficient to realize GTPase activity (26, 28). We suggest that κB-Ras provides a scaffold that utilizes these mechanisms, leading to the observed intrinsic hydrolysis activity. Possibly, similar features also mediate the intrinsic GTP hydrolysis of other GTPases that do not require a catalytic glutamine, like Rheb or Rap (10, 12).

## Experimental procedures

### Protein purification

Murine glutathione-*S*-transferase (GST)-RalA G-domain (residues 9–183) and human GST-κB-Ras1 (residues 2–175) were expressed in *Escherichia coli* BL21 and Rosetta, respectively, purified by affinity chromatography with GSH-Agarose beads (Serva), followed by on-column cleavage with PreScission protease. Recombinant κB-Ras1–RGα2N was obtained by coexpression of GST-κB-Ras1 (2–175) and 6xHis-SUMO RGα2N (2–255) and purified essentially as described previously, but with the addition and incubation (3 h) of SUMO protease. Samples were further purified with a Superdex 75 pg 16/600 (GE Healthcare) or ENrich SEC70 (Bio-Rad) size-exclusion column. RalGAP complexes were expressed in Expi293F cells and purified essentially as described (21).

### Structure determination

The κB-Ras1/RGα2N complex supplemented with 100 μM GNP was crystallized with seeds from initial hits in a hanging drop vapor diffusion setup at 4 °C with a reservoir solution containing 0.1 M magnesium acetate, 17% w/v PEG3350, and 20% v/v glycerol. Diffraction data were collected at EMBL beamline P14 (PETRA III) at 0.68879 Å in helical collection mode. A 2.72 Å dataset (Table S1) was processed with autoProc (Global Phasing) (29) and STARANISO (Global Phasing) (30). Trimmed AlphaFold2 (31) models of the individual subunits were used for molecular replacement in phaser_MR (32). The final model was manually built in COOT (MRC Laboratory of Molecular Biology) (33) and refined with phenix.refine using translation/rotation/screw-rotation refinement (34). Figures were prepared with ChimeraX (UCSF) (35) and ESPript3 (36).

### GTPase activity assays

Protein samples were mixed with 20 mM EDTA, 1 mM DTT, and 5 mM MgCl_2_ in assay buffer C (30 mM Hepes [pH 7.5] and 150 mM NaCl). Reactions were started by addition of 50 μM (GAP stimulated) or 100 μM (intrinsic) GTP. GTP–GDP ratios of samples taken after 0 and 60 min (stimulated) or 24 h (intrinsic) were determined by HPLC analysis on an AMAZE HA mixed phase column (Helix Chromatography).

### Coprecipitation

Human embryonic kidney 293FT cells were transfected with p3xFLAG-CVM7-RGα2, HA-pKH3-RGβ, and pCTAPa-κB-Ras1, respectively, and lysed with co-IP buffer (40 mM Hepes, 120 mM NaCl, 10 mM MgCl_2_, 0.3% CHAPS, pH 7.4, protease inhibitors [Serva; 1:100 dilution]). Streptavidin Sepharose High Performance (20 μl; Cytiva) were incubated with the cleared lysate, washed, and resuspended in 1x SDS-loading dye for immunoblot analysis.

### Generation and reconstitution of RG**α**2 knockout murine embryonic fibroblasts

For CRISPR–Cas9 knockout (34), single guide RNAs targeting the ATG-containing exon 1 of *RALGAPA2* were designed with the CRISPick tool (37, 38) and cloned into px458 or px459 plasmids (39). SV40-immortalized WT murine embryonic fibroblasts (MEFs) (18) were transfected with the plasmids, and GFP-positive cells were fluorescence-activated cell sorted 24 h after transfection and selected with 2 μg/ml puromycin for 36 h. Single-cell clones were obtained by limited dilution, and deletion of RGα2 was confirmed by PCR screening, RT–quantitative PCR, and immunoblot. RGα2 or RGα2Δloop were cloned into pITR-TTP (40) with N-terminal ALFA tag and transfected into RGα2KO MEFs. Cells were selected with puromycin, and exogenous protein expression was confirmed by immunoblot.

### Ral effector pull down

WT, RGα2KO, and reconstituted RGα2KO MEFs were lysed in 800 ml Ral lysis buffer (50 mM Tris–HCl, 100 mM NaCl, 4 mM MgCl_2_, 2 mm EGTA, 1% Triton X-100, pH 7.5 at 4 °C). The Ral-binding domain of Sec5 was purified from *E. coli* as GST-fusion construct and loaded onto GSH beads. GST-Sec5 (20 μg) was incubated with cleared lysate, washed, and resuspended in 1x SDS loading dye.

### Antibodies

The following antibodies were used for immunoblotting: α-RalA (1:1000 dilution; Proteintech, catalog no.: 13629-1-AP), α-RalB (1:1000 dilution, OTI2C4; Origene, catalog no.: TA505880), α-κB-Ras (1:1000 dilution, provided by S. Ghosh, Columbia University (18)), α-ALFA (1:2000 dilution; Nanotag, catalog no.: N1582), α-FLAG (1:2000 dilution; clone M2; Sigma–Aldrich, F1804), α-HA (1:2000 dilution, clone 16B12; BioLegend), α-mouse-horseradish peroxidase (1:10,000 dilution, P0260; Dako), α-rabbit-horseradish peroxidase (1:10,000 dilution, P0217; Dako), α-mouse-IRDye800CW (1:4000 dilution, 926-32212; LI-COR), and α-rabbit-IRDye680RD (1:4000 dilution, 926-68073; LI-COR).

## Data availability

Protein structure coordinates and experimental structure factors have been deposited in the Protein Data Bank (ID: 9QU1). All other data required to assess the conclusion of the study are included in the article. Original data are available on reasonable request.

## Supporting information

This article contains supporting information (18, 21, 29, 30, 31, 32, 33, 34, 37, 38, 39, 40).

## Conflict of interest

The authors declare that they have no conflicts of interest with the contents of this article.

## Supporting information

Supplemenatary Material
