# Supplementary material for: The GTPase κB-Ras is an essential subunit of the RalGAP tumor suppressor complex
Source: J Biol Chem. 2025 Jul 5;301(8):110460. doi: 10.1016/j.jbc.2025.110460 (PMC12314375; doi:10.1016/j.jbc.2025.110460)
Supplement: Supplemenatary Material [file mmc1.pdf]

Table S1: Data collection and refinement statistics for the structure determination of the κB-Ras1/RGα2-N crystal structure.

**Data Collection**	PDBID 9QU1
Space Group	C 1 2 1
Unit-cell parameters	
a, b, c (Å)	163.6, 63.4, 104.2
α, β, γ (°)	90, 91.3, 90
Resolution range (Å) [1]	63.63 - 2.72 (2.84 - 2.72)
Observed reflections[1]	121357 (4981)
Unique reflections[1]	21829 (1093)
Ellipsoidal completeness (%)[1,2]	91.5 (57.9)
Spherical completeness (%)[1]	74.9 (7.6)
Multiplicity[1]	5.6 (4.6)
Wilson B-factor	65.5
I/σ(I) [1]	10.2 (1.2)
$CC_{1/2}$[1]	0.997 (0.508)
R_{meas} (%)[1]	12.5 (136.3)
**Refinement**	
Resolution range (Å) [1]	63.6 - 2.72 (2.84 - 2.72)
R_{work} / R_{free} (%)[1]	19.5 / 25.3 (26.8 / 40.58)
Reflections [1]	21829 (274)
Ramachandran favored (%)	98.68
Ramachandran allowed (%)	1.32
Ramachandran outliers (%)	0.0
RMS bonds (Å)	0.004
RMS angles (°)	0.64
Average B-factor	75.5
macromolecules	75.7
ligands	70.3
Number of TLS groups	20

[1] Numbers in parenthesis refer to outer shell of reflections

[2] ellipsoidal truncation and scaling with STARANISO, diffraction limits a = 3.1 Å, b= 3.0 Å, c = 2.6 Å

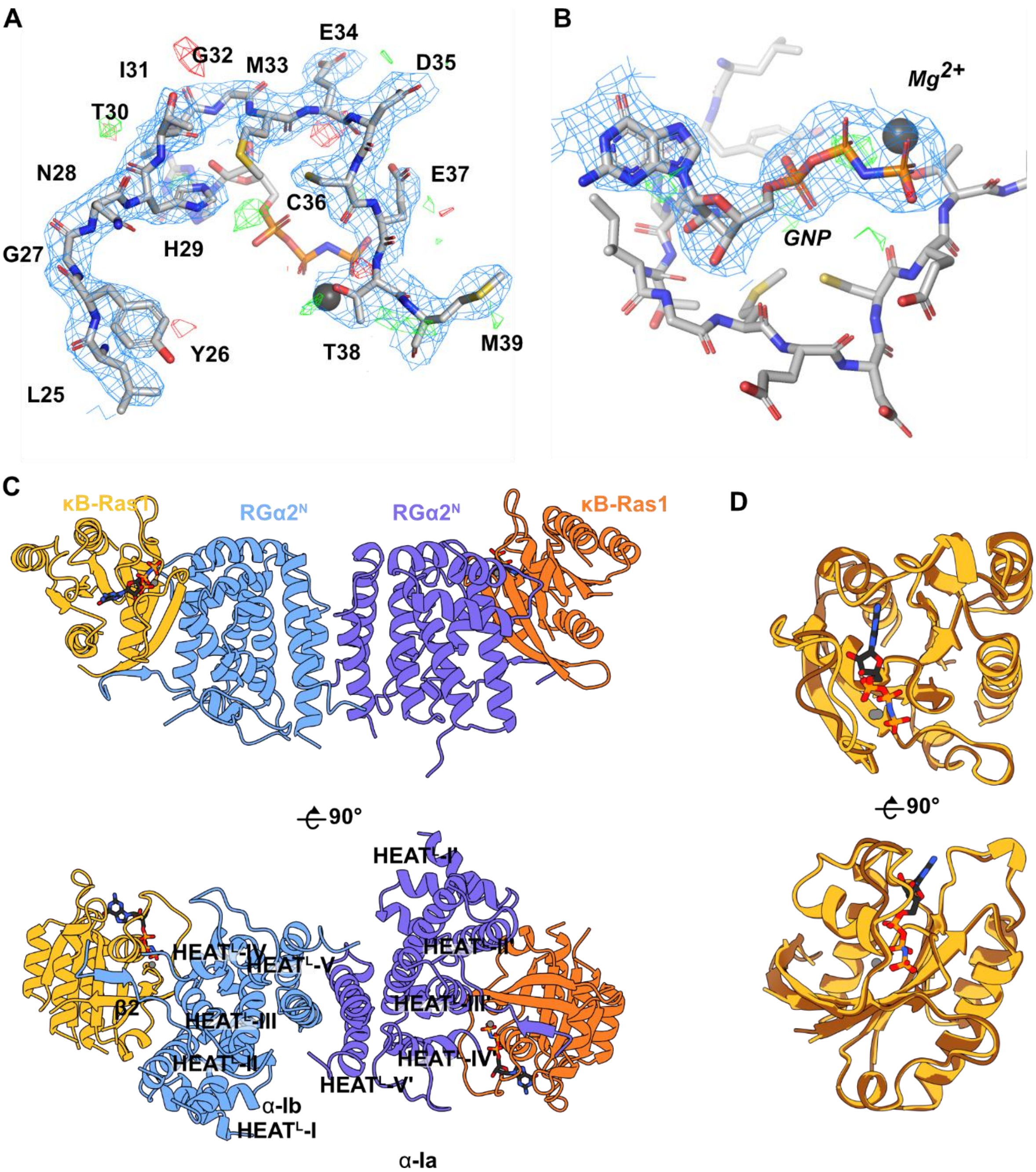

Figure S1: Crystal structure of the κB-Ras1/RGα2-N complex. Close-up of the (A) switch I region of κB-Ras1 and (B) the bound GNP nucleotide with a $2F_o$-F_c electron density map (blue, contoured at 1 σ level) and an F_o-F_c difference electron density map (colored green and red at +3 and -3 σ level, respectively).(C) Two κB-Ras1/RGα2-N complexes interact tail-to-tail via the truncated end of the RGα2-N α solenoid in the asymmetric unit. (D) Superposition of the two κB-Ras1 copies (yellow and brown) in the asymmetric unit shows different switch I conformation.

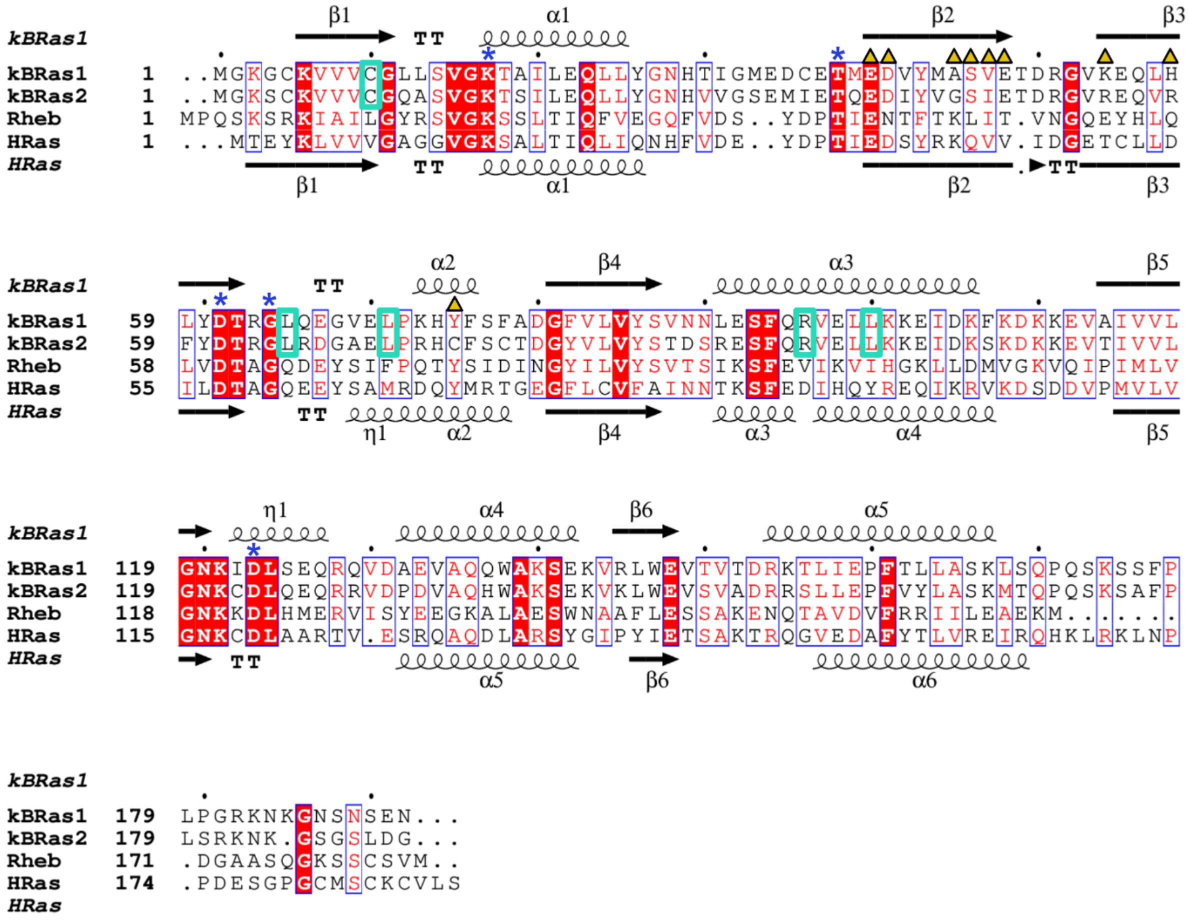

Figure S2: Structure-based multiple sequence alignment of κB-Ras1, κB-Ras2, Rheb, and H-Ras. Key residues for nucleotide interaction are labeled with a blue asterisk and residues that stabilize the κB-Ras switch II conformation are marked by a green box. RG2αN-interacting residues of κB-Ras1 are indicated by an orange triangle.

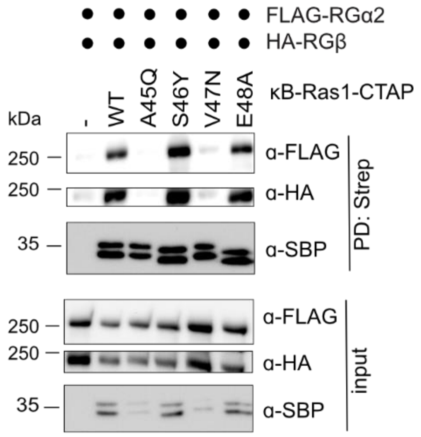

Figure S3: Co-immunoprecipitation of RGα2/β by κB-Ras1 wild-type and variants.

Supplementary experimental information

Molecular Biology

For cell culture experiments, human RalGAPα2 and RalGAPβ were cloned into a 3xFLAG-pCVM7.1 and HA-pKH3 vector, respectively, and κB-Ras1 DNA sequence was cloned into pCTAPa vector (C-terminal tandem Strep binding peptide (SBP) and calmodulin binding peptide (CBP) tags). Murine RalA G-domain (residues 9-183) and human κB-Ras1 (residues 2-175) were cloned into pCDF6P. RalGAPα2^N (residues 2-255) was cloned into pET28 with an N-terminal 6xHis-SUMO (small ubiquitin-like modifier) tag. Mutations were generated using the Q5 Site-Directed Mutagenesis Kit (New England Biolabs).

Protein expression and purification

BL21 Rosetta *Escherichia coli* were transformed with κB-Ras1 or κB-Ras2 constructs and grown in TB media to an OD_{600nm} of 0.9-1.0. Expression of the GST-tagged protein was induced by adding IPTG (0.5 mM f.c.) and conducted overnight at 16 °C. Cell pellets from a 2 l culture were resuspended in buffer L (50 mM NaH_2PO_4 pH 8.0, 300 mM NaCl, 1 mM $MgCl_2$, 1 mM DTT, 5 % glycerol), supplemented with lysozyme, DNaseI and protease inhibitor cocktail (Mix HP, Serva) and lysed (Microfluidizer). The lysate was cleared by centrifugation (39 000 x*g*, 45 min, 4 °C). The supernatant was loaded onto equilibrated GSH-Agarose beads (Serva) three times and washed three times with 20 ml buffer L. GTP was added to a final concentration of 100 µM and the protein was cleaved from the beads with PreScission protease overnight. The supernatant was collected, and the protease was removed by passing the eluate over Ni-NTA beads (Serva). The eluate was concentrated (Amicon MWCO = 10 000 Da, steps of 3 min, 3 900 x*g*) to approximately 500 µl and loaded onto an ENrich™ SEC70 size exclusion column (BioRad) equilibrated with buffer A (30 mM HEPES pH 7.4, 300 mM NaCl, 1 mM $MgCl_2$, 1 mM TCEP, 5 % glycerol). Peak fractions were collected and concentrated to 40 – 120 µM. Murine RalA G-domain was expressed and purified likewise except for the addition of GTP. A Superdex75 pg 16/600 HiLoad column (GE Healthcare) used for size exclusion chromatography and peak fractions concentrated to 800 µM, flash- frozen aliquots stored at -70 °C upon use.

Recombinant κB-Ras1:RGα2N was obtained by co-expression of pCDF6P κB-Ras1 2-175 and pET28 6xHis-SUMO RGα2N 2-255 in BL21 *E. coli* and purified essentially as described above, but with the addition and incubation (3 h) of SUMO protease before the last washing step. Samples were loaded on a Superdex™ 75 pg 16/600 column (GE Healthcare) equilibrated with buffer B (20 mM HEPES pH 7.4, 200 mM NaCl, 2 mM TCEP, 2 mM $MgCL_2$, 1.5 % glycerol). RalGAP complexes were expressed in Expi293F cells and purified essentially as described (1).

Crystallization

The purified κB-Ras1:RGα2N complex was supplemented with 100 µM non-hydrolysable GTP analog 5'-guanylylimidodiphosphate (GNP) to ensure a homogenous nucleotide loading state during crystallization trials, which were performed with the sparse matrix sitting drop vapor diffusion method. Reservoir solution and protein were dispended by a ARI Gryphon pipetting robot on Swissci MRC plates. Initial microcrystal hits were found in different conditions from JSCG+ and proPlex screens (Molecular Dimensions) containing PEG3350 or PEG4000 and magnesium salts. Crystal quality was iteratively improved in optimization rounds and using the initial hits as crystallization seeds. Rod-like crystals were grown at 4 °C from a condition containing 0.1 M magnesium acetate, 17% w/v PEG3350 and 20% v/v glycerol at 4 °C in a hanging drop vapour diffusion set-up. Crystals were harvested with nylon loops after 17 days and flash cooled in liquid nitrogen.

Structure Determination

Diffraction data was collected at EMBL P14 beamline at PETRA III light source at 0.68879 Å. Due to the elongated crystal shape, data were helically collected. A dataset from a single crystal was processed by the autoProc (2) pipeline and curated by the STARANISO (3) protocol to correct for anisotropy. The phenix software suite was used for further processing (4). For phases determination, trimmed AlphaFold2 (5) models of the individual subunits were used for molecular replacement in phaser_MR (6). The initial structure model was manually corrected in COOT (7) and refined in phenix.refine using TLS refinement.

GTPase activity assays

GTPase activity was assayed as previously described (1). For intrinsic activity, 30 µM K-Ras, Rheb orκB-Ras1 and 100 µM GTP were used. For stimulated activity, RalA (10 µM) and RalGAP complex (0.4 µM) were mixed with 50 µM GTP. Proteins were mixed with 20 mM EDTA, 1 mM DTT and 5 mM $MgCl_2$ in assay buffer C 30 mM HEPES pH 7.5, 150 mM NaCl. GTP was added to start the reaction and aliquots were snap frozen in liquid nitrogen after 0 min and 60 min (24 h for intrinsic activity).

To analyze nucleotide ratios, samples were boiled (5 min, 95 °C) and precipitated protein removed by centrifugation (20 min, 21 000 g,4 °C). Ten microliters of the clear supernatant were loaded with an HPLC system (Agilent 1260 Infinity, Agilent, Santa Clara, CA, USA) on a AMAZE HA mixed phase column (Helix Chromatography, particle size 3 µm, pore size 100 Å, stainless steel column 50 × 3 mm) applying a double step gradient method with buffer A (200 mM K_2HPO_4, pH 2.0; 30-80 %) and acetonitrile (15-20 %). The UV absorbance at 254 nm was used to monitor nucleotide elution. UV traces were semi-automatically analyzed with OriginPro

2024 (OriginLab Corporation). Traces were base line corrected, the peaks corresponding to GDP and GTP integrated and the portion of GTP calculated. The remaining GTP amount was normalized to GTP at t = 0 h. Individual biological repeats were calculated from three technical replicates.

Cell lines

HEK293FT (RRID:CVCL_6911) cells were obtained from Thermo Fisher Scientific (R70007). Mouse embryonic fibroblasts (MEFs) were generated from a C57BL/6 mouse embryo at day E12.5 and SV40-immortalized (8). CRISPR/Cas9 mediated knockout of *RalGAPA2* was performed as described below (9). All cell lines were cultured in DMEM high glucose (Sigma-Aldrich, D6546) supplemented with 10 % FBS (Biowest, S1810), 10 mM L-Glutamine (Sigma-Aldrich, G7513) and 50 U Penicillin-Streptomycin (Thermo Fisher Scientific, 15070063) at 37 °C with 5 % CO_2.

CRISPR/Cas9-based generation of RalGAPα2 knockout MEFs

For CRISPR knockouts sgRNA sequences were chosen with the CRISPick tool (10, 11). Annealed Oligos were cloned into px458 or px459 plasmids (gifts from Feng Zhang; Addgene plasmids # 48138 and # 62988(9)). SV40-immortalized WT MEFs were simultaneously transfected with px458 and px459 plasmids containing gRNAs targeting upstream (RGα2sg1 tcaggcacagaggttacccg) and downstream (RGα2sg2 gcggaccagaggcacccgtg) of the ATG-containing exon1 of RGα2 using jetPRIME® DNA and siRNA transfection reagent (Polyplus, 101000046). 24 h after transfection GFP-positive cells were FACS-sorted, returned to culture to recover overnight, and then selected for 36 hours with 2 µg/ml puromycin. Single cell clones were obtained by limited dilution. Successful deletion of RGα2 was tested using PCR screening (mRGα2 screen 4F gagtgagagatggtagttcaacg, mRGα2 screen 4R ggaggtcactgcgcagactcgaagc) and confirmed by RT-qPCR using primers targeting inside the deleted exon (mRGα2RTEx1_F gaaggagccacggagatgt, mRGα2RTEx1_R catccacgttatccagcagc) and immunoblot.

Reconstitution of RalGAPα2 knockout MEFs

N-terminally ALFA-tagged RGα2 or RGα2$^{\Delta loop}$ were cloned into a modified pITR-TTP vector (12) via MluI and NotI restriction sites. RGα2KO MEFs were transfected with the plasmids and the transposase expressing pCMV-Trp plasmid (9:1 ratio) using jetPRIME DNA and siRNA transfection reagent (Polyplus, 101000046) for stable reconstitution. Cells were selected for 48 hours with 2 µg/ml puromycin and maintained with 1.5 µg/ml puromycin afterwards. To confirm exogenous expression, proteins were isolated with Bäuerle lysis buffer (20 mM Tris pH 8, 350 mM NaCl, 20 % glycerine, 1 mM MgCl2, 0.5 mM EDTA, 0.1 mM EGTA, 1 % NP-40

supplemented with 1 mM DTT, Protease and Phosphatase Inhibitor Cocktail (Thermo Fisher Scientific, A32965, A32957) and analyzed by immunoblot.

Ral effector pull down

WT, RGα2KO, and reconstituted RGα2KO MEFs were lysed in 800 ml SLB (50 mM Tris-HCl, 100 mM NaCl, 4 mM MgCl2, 1 % Triton-X100, pH 7.5 at 4 °C). The Ral-binding domain of rSec5 was purified from *E. coli* as GST-fusion construct and loaded onto GSH beads. 20 µg GST-rSec5 were added to the cleared cell lysate and incubated (45 min, 4 °C, 5 rpm). The supernatant was removed and the beads washed with SLB three times before resuspending in 1x SDS loading dye.

Co-Immunoprecipitation

HEK293FT cells were seeded to 10 cm petri dishes and grown to 70 % confluency. The medium was exchanged to starvation medium and incubated with DNA-PEI mixture for 4 h at 37 °C 5 % CO2. Medium was changed to growth medium. The next day, medium was removed and the cells washed 1-2 times with ice cold PBS. 1 ml CoIP buffer (40 mM HEPES, 120 mM NaCl, 10 mM MgCl2, 0.3 % CHAPS, pH 7.4) supplemented with PIC (1:100) was added to the cells and cells collected using a gum wiper. Cells were incubated for 20 min on a stirring wheel at 4 °C before debris was removed by centrifugation. The cleared lysate was incubated with 20 µl 3xflag (Sigma) or Streptavidin Sepharose High Performance (cytiva) beads slurry for 2-4 h. Beads were pelleted, the supernatant removed, and the beads washed three times with CoIP buffer. Beads were resuspended in 1 x SDS-LD and analyzed by western blotting.

Immunoblot

Proteins were separated by SDS-PAGE and transferred to Immobilon-FL (Millipore, IPFL00010) membrane using a semidry system blotting system (Bio-rad). For enhanced chemiluminescence (ECL) detection, membranes were blocked in 3 % BSA/ TBS (α-SBP) or 5 % dry-milk powder/ TBS (α-FLAG M2, α-HA, α-κB-Ras) for 1 h at RT. Primary antibodies were diluted in TBS and incubated with the membranes overnight at 4 °C. The membranes were incubated with the secondary antibody in TBS at RT for 1.5 h. Detection was performed with the SuperSignal West Pico PLUS detection reagent kit (Thermo Scientific, Ref 34577) on an Intas ECL Chemostar system. For imaging on the Odyssey CLx Imaging System (LI-COR), membranes were blocked for 1 h in Blocking Buffer (0.1 % casein (Sigma-Aldrich, E0789) in 0.2 x PBS). Primary antibodies were diluted 1:1000 in 1:1 PBS:Blocking Buffer with 0.1 % Tween and incubated with the membranes at 4 °C overnight. Secondary antibodies were applied 1:4000 for 1 h in 1:1 PBS:Blocking Buffer with 0.1 % Tween and 0.01 % SDS.

The following antibodies were used: α-RalA (1:1000, Proteintech, 13629-1-AP), α-RalB (1:1000, OTI2C4, Origene, TA505880), α-κB-Ras (1:1000, provided by S. Ghosh, Columbia

University(8)), α-ALFA (1:2000, Nanotag, N1582), α-FLAG (1:2000, clone M2, Sigma-Aldrich, F1804), α-HA (1:2000, clone 16B12, Biolegend), α-mouse-HRP (1:10000, P0260, Dako), α-rabbit-HRP (1:10000, Dako, P0217), α-mouse-IRDye800CW (1:4000, LI-COR, 926-32212), α-rabbit-IRDye680RD (1:4000, LI-COR, 926-68073).

Validations of the antibodies purchased from commercial vendors are available on the manufactures' websites and/or datasheets sent to us. RalA and RalB antibodies were further validated by testing on immunoblots with lysates from respective knock-down cell lines. The specificity of the kB-Ras antibody in immunoblots was tested using both mouse and human knockout cells.

Statistical Analysis

Data curation and statistical analysis were done with OriginPro 2024 (OriginLab Corporation). Normality was tested with a Shapiro-Wilk test and significance was determined with one-sided ANOVA test and Bonferroni correction.

Supplementary references

1. Rasche, R., Klink, B. U., Apken, L. H., Michalke, E., Chen, M., Oeckinghaus, A., Gatsogiannis, C., and Kümmel, D. (2024) Structure and mechanism of the RalGAP tumor suppressor complex. *bioRxiv*. 10.1101/2024.11.25.625123

2. Vonrhein, C., Flensburg, C., Keller, P., Sharff, A., Smart, O., Paciorek, W., Womack, T., and Bricogne, G. (2011) Data processing and analysis with the autoPROC toolbox. *Acta Crystallogr D Biol Crystallogr*. 67, 293–302

3. Tickle, I. J., Flensburg, C., Keller, P., Paciorek, W., Sharff, A., Vonrhein, C., and Bricogne, G. (2016) STARANISO. *Cambridge, United Kingdom: Global Phasing Ltd*

4. Adams, P. D., Afonine, P. V, Bunkóczi, G., Chen, V. B., Davis, I. W., Echols, N., Headd, J. J., Hung, L.-W., Kapral, G. J., Grosse-Kunstleve, R. W., McCoy, A. J., Moriarty, N. W., Oeffner, R., Read, R. J., Richardson, D. C., Richardson, J. S., Terwilliger, T. C., and Zwart, P. H. (2010) PHENIX: a comprehensive Python-based system for macromolecular structure solution. *Acta Crystallogr D Biol Crystallogr*. 66, 213–21

5. Jumper, J., Evans, R., Pritzel, A., Green, T., Figurnov, M., Ronneberger, O., Tunyasuvunakool, K., Bates, R., Žídek, A., Potapenko, A., Bridgland, A., Meyer, C., Kohl, S. A. A., Ballard, A. J., Cowie, A., Romera-Paredes, B., Nikolov, S., Jain, R., Adler, J., Back, T., Petersen, S., Reiman, D., Clancy, E., Zielinski, M., Steinegger, M., Pacholska, M., Berghammer, T., Bodenstein, S., Silver, D., Vinyals, O., Senior, A. W., Kavukcuoglu, K., Kohli, P., and Hassabis, D. (2021) Highly accurate protein structure prediction with AlphaFold. *Nature*. 596, 583–589

6. McCoy, A. J., Grosse-Kunstleve, R. W., Adams, P. D., Winn, M. D., Storoni, L. C., and Read, R. J. (2007) Phaser Crystallography Software. *J. Appl. Chrystallog.* 40, 658–674

7. Emsley, P., Lohkamp, B., Scott, W. G., and Cowtan, K. (2010) Features and development of Coot. *Acta Crystallogr D Biol Crystallogr*. 66, 486–501

8. Oeckinghaus, A., Postler, T. S., Rao, P., Schmitt, H., Schmitt, V., Grinberg-Bleyer, Y., Kühn, L. I., Gruber, C. W., Lienhard, G. E., and Ghosh, S. (2014) κB-Ras proteins regulate both NF-κB-dependent inflammation and Ral-dependent proliferation. *Cell Rep*. 8, 1793–1807

9. Ran, F. A., Hsu, P. D., Wright, J., Agarwala, V., Scott, D. A., and Zhang, F. (2013) Genome engineering using the CRISPR-Cas9 system. *Nat Protoc*. 8, 2281–2308

10. JG, D., N, F., M, S., M, H., EW, V., KF, D., I, S., Z, T., C, W., R, O., HW, V., J, L., and DE, R. (2016) Optimized sgRNA design to maximize activity and minimize off-target effects of CRISPR-Cas9. *Nat Biotechnol*. 10.1038/NBT.3437

11. Sanson, K. R., Hanna, R. E., Hegde, M., Donovan, K. F., Strand, C., Sullender, M. E., Vaimberg, E. W., Goodale, A., Root, D. E., Piccioni, F., and Doench, J. G. (2018) Optimized libraries for CRISPR-Cas9 genetic screens with multiple modalities. *Nat Commun*. 9, 5416

12. Fitzian, K., Brückner, A., Brohée, L., Zech, R., Antoni, C., Kiontke, S., Gasper, R., Linard Matos, A. L., Beel, S., Wilhelm, S., Gerke, V., Ungermann, C., Nellist, M., Raunser, S., Demetriades, C., Oeckinghaus, A., and Kümmel, D. (2021) TSC1 binding to lysosomal PIPs is required for TSC complex translocation and mTORC1 regulation. *Mol Cell*. 81, 2705–2721
